# A deep learning algorithm for automated measurement of vertebral body compression from X-ray images

**DOI:** 10.1038/s41598-021-93017-x

**Published:** 2021-07-02

**Authors:** Jae Won Seo, Sang Heon Lim, Jin Gyo Jeong, Young Jae Kim, Kwang Gi Kim, Ji Young Jeon

**Affiliations:** 1 Department of Health Sciences and Technology, GAIHST, Gachon University, Incheon, 21999 Korea; 2 Department of Biomedical Engineering, Gachon University College of Medicine, 38-13 Docjeom-ro 3-bungil, Namdong-gu, Incheon, 21565 South Korea; 3 Department of Radiology, Gachon University College of Medicine, Gil Medical Center, 38-13 Docjeom-ro 3-bungil, Namdong-gu, Incheon, 21565 South Korea

**Keywords:** Biomedical engineering, Electrical and electronic engineering, Health care, Medical research

## Abstract

The vertebral compression is a significant factor for determining the prognosis of osteoporotic vertebral compression fractures and is generally measured manually by specialists. The consequent misdiagnosis or delayed diagnosis can be fatal for patients. In this study, we trained and evaluated the performance of a vertebral body segmentation model and a vertebral compression measurement model based on convolutional neural networks. For vertebral body segmentation, we used a recurrent residual U-Net model, with an average sensitivity of 0.934 (± 0.086), an average specificity of 0.997 (± 0.002), an average accuracy of 0.987 (± 0.005), and an average dice similarity coefficient of 0.923 (± 0.073). We then generated 1134 data points on the images of three vertebral bodies by labeling each segment of the segmented vertebral body. These were used in the vertebral compression measurement model based on linear regression and multi-scale residual dilated blocks. The model yielded an average mean absolute error of 2.637 (± 1.872) (%), an average mean square error of 13.985 (± 24.107) (%), and an average root mean square error of 3.739 (± 2.187) (%) in fractured vertebral body data. The proposed algorithm has significant potential for aiding the diagnosis of vertebral compression fractures.

## Introduction

Vertebral compression fractures account for most vertebral fractures^1^, with approximately 1.5 million vertebral compression fractures occurring annually in the United States^2^. Many studies have been conducted on osteoporotic vertebral compression fractures, which account for the largest percentage of vertebral compression fractures^3^.

The treatment of vertebral compression fractures varies according to the type of fracture, or Kyphotic angulation measured on plain lateral radiographs. If initial vertebral height loss is measured to be over 40% and fracture kyphosis is measured to be over 30°, then operative treatment is generally indicated^4^. If conservative management selected, the patient is serially followed for progression of deformity. Significant progression (magnitude undefined) on the vertebral height loss or kyphotic angulation is often considered a conservative treatment failure. Therefore, reliable and reproducible radiographic measurements are essential for clinical decision making. There are various radiographic measurement parameters used to vertebral compression fractures on lateral radiographs such as Cobb angle, vertebral compression ratio, and anterior vertebral body compression percentage (Eq. 1)^5–7^. Anterior vertebral body compression percentage (VC) is the percentage of decrease in the height of a vertebral body^8,9^. As these are done manually by observers, variability in the measurement value is bound to occur even if the same methods are used. There is the effect of the technical quality of the radiograph and the subsequent ability of the clinician to interpret it, which is encompassed by the intraobserver and interobserver variability. Therefore, manual measurement increases the likelihood of misdiagnosis, inter-observer variability, and delayed diagnosis, which can be fatal for the patient^9,10^. Consequently, there have been studies on various methods for overcoming these shortcomings^11,12^.

Artificial intelligence (AI) has become distinguished in medical imaging and computer vision and has demonstrated positive results and exceptional performance in medical imaging applications across multiple studies^13^. The convolutional neural network (CNN), a deep learning AI algorithm, has a generalized performance with higher precision than existing image processing technology and provides excellent performance in terms of efficiency when applied to medical images^13–15^.

Therefore, there has recently been a considerable amount of research on using deep learning to assist spinal disease diagnosis. Some studies proposed deep learning models based on CNN for segmentation vertebrae^16–20^. Some researchers proposed a cascade amplifier regression network (CARN) based on a CNN for estimating vertebral body height and intervertebral disc height in MR images^21^. While these studies have demonstrated promising performances and various other studies have indicated that deep learning is suitable for diagnosing spinal disease, to the best of our knowledge, this is the first time to directly measure the VC using a CNN.

This study proposes an algorithm that automatically segments the vertebral bodies and measures the VC from the spine X-ray image using a CNN-based model, thus overcoming the shortcomings of manual VC measurement. Our deep learning tool for automated measurement of VC could minimize the observer variability in comparison with manual measurement, which can be the superior method in terms of cost-effectiveness, reliability and reproducibility.

## Materials and methods

**Figure 1:**
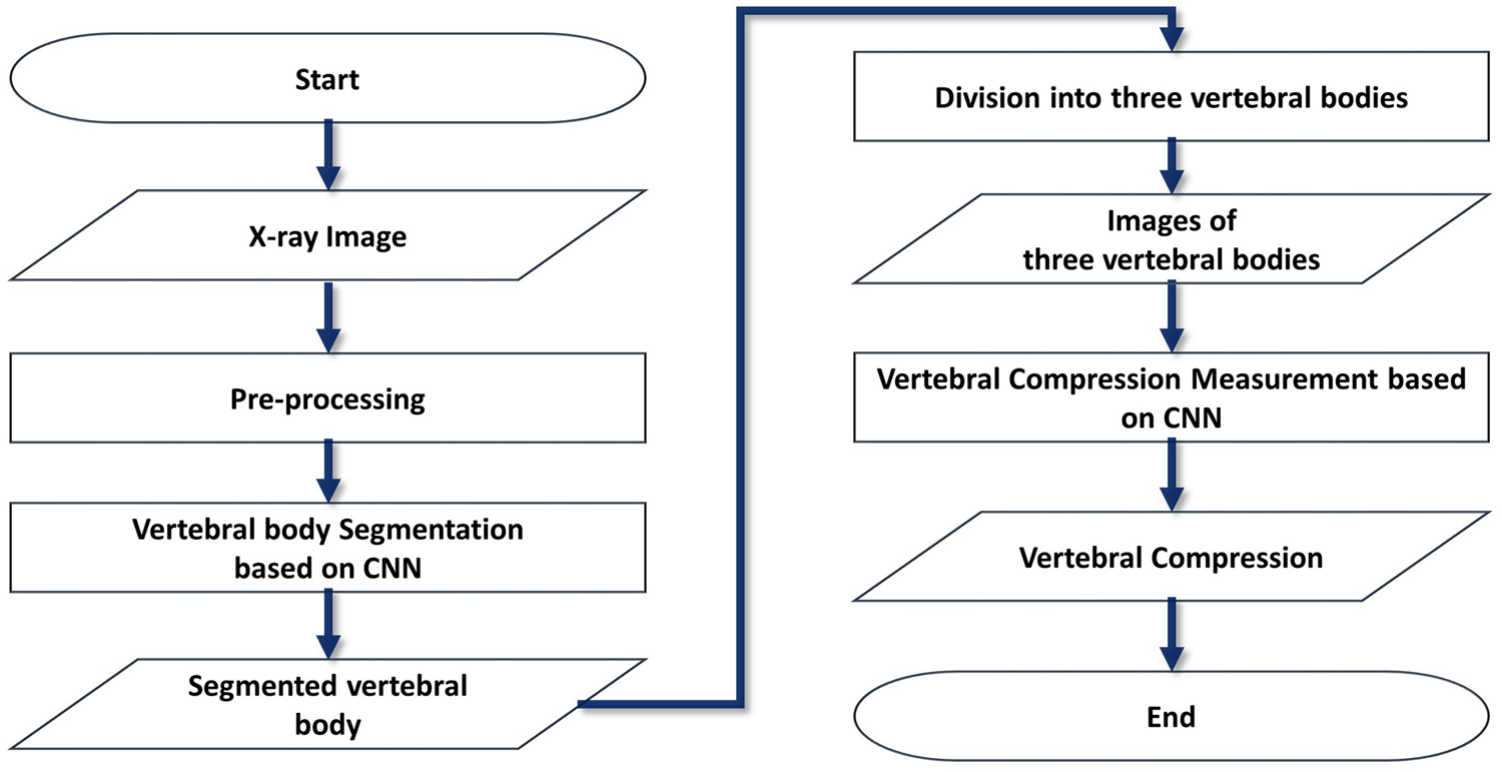
Flow chart of the proposed vertebral compression (VC) measurement process.

The proposed method involves using a segmentation and regression CNN to measure the VCR. A flow chart of the process is presented in Fig. 1.

We preprocessed lateral X-ray images of the vertebrae and segmented vertebral bodies (VBs) from the preprocessed images using a vertebral body segmentation model. Three VBs were then separated from the segmented VB images and used as input values for the VC measurement model. Finally, the model delivered a measured VC.

### Experiment setup

The CNNs were implemented in Python 3.6.10 using Keras 2.2.5 frameworks on an Ubuntu 14.04 operating system and trained on a workstation equipped with four NVIDIA RTX 2080Ti GPUs and 128 GB of RAM.

### Data acquisition and preprocessing

This study was performed as a retrospective study with permission from the Institutional Review Board of the Gil Medical Center (IRB number: GDIRB2019-137). The informed consent was obtained from all patients at this institution. All experimental protocols were performed in accordance with the relevant guidelines and regulations in compliance with the Declaration of Helsinki. 387 thoracic and lumbar spine lateral X-ray images with vertebral compression fractures from the 300 subjects were included for this study. All of these patients had vertebral compression fractures and treated by the orthopedic and neurosurgery department. As it was anonymized data, only few of patient's demographic characteristics could be identified. Patients ranged in age from 28 to 86 years, and the mean age was 59 years. Fractures were at T8-L5. A radiologist manually generated the ground truth for the segmentation. 387 lateral radiographs of thoracic and lumbar fractures were measured by a board-certified musculoskeletal radiologist with 10 years’ experience; Intraobserver Reliability: the intraclass coefficient varied from 0.70 to 0.91 for the reader.

Because of the variation in X-ray image size and pixel spacing between patients, we applied contrast-limited adaptive histogram equalization (CLAHE) image processing to enhance the local contrast for improving segmentation of vertebral bodies (Fig. 6b) and a zero padding to set the image size to a standardized 512 × 512 pixels based on the original ratio.

We divided the collected 387 images into 323 and 64, about 5:1 ratio, and used 323 in the model for vertebral body segmentation based on CNN. The remaining 64 that were not used for training were used for performance evaluation. Because calculation the anterior vertebral compression needs three of anterior vertebral height, as shown in Fig. 4, we divided the segmented VBs from segmentation model into three using image processing except for 83 images that failed to segment. The segmentation result that satisfies the following three conditions was defined as "accurate segmentation results". First, each vertebral body should be subdivided into one. Second, the corners of vertebral bodies should be able to be found. Third, in order to use the Eq. (1) for calculating vertebral compression, the three vertebral bodies should be adjacent to each other and divided. Therefore, vertebral compression measurement methods were applied except for the results that do not correspond to the three conditions (see Supplementary Fig. S1 online). From the process, we generated 1366, three VB images from segmented VBs of the 304 images. The 945 data of 1134 data were used for training vertebral measurement model and remained 189 were used for evaluating performance of model.

### Vertebral body segmentation model based on CNN

Because vertebral compression measurements are affected by the results of VB segmentation, high accuracy segmentation is required. U-net, developed mainly for medical image analysis, has the advantage of being able to precisely segment an image by using an insufficient amount of training data^22–24^. Therefore, we applied our data to U-net, residual U-net (ResU-net) with residual block applied to this U-net, and a recurrent residual U-Net (R2U-Net) with recurrent process added to ResU-net^25^. Performance evaluation was performed using test data not used for training, and R2U-net, which showed the highest performance, was selected based on the dice similarity coefficient among the three models.

R2U-Net shows excellent performance in medical image segmentation when compared with other CNNs and is composed of a residual unit and a recurrent CNN model in which several convolution operations share one kernel weight and perform multiple iteration operations. Therefore, it has the advantage of improving the expression of a feature value by adding the input value to the output value of the corresponding layer via an element-wise operation, enabling deep structure learning and accumulating feature values.

The model is constructed four encoders and decoders comprising a recurrent convolution 2D filter (Recurrent Conv2D) with the time step set to two, batch normalization (BN), and an activation function rectified linear unit (ReLU). The encoder has four layers and comprises the Recurrent Conv2D with a 3 × 3 kernel size and a 1 × 1 stride, BN, and the ReLU. The encoder captures context and reduces the size of the feature map via max pooling with a 2 × 2 kernel size and a 2 × 2 stride per layer. The decoder consists of four layers and comprises a recurrent upsampling convolution 2D (Recurrent Up-Conv2D) layer, BN, and ReLU. The decoder prevents spatial information loss by upsampling the feature map with a 2 × 2 size and concatenates the neural network used in the encoder. The segmentation map was extracted using Recurrent Conv2D, 1 × 1 convolution, and a sigmoid activation function. To use the ground truth (GT) for training, specialists manually segmented VBs. The model was trained for a batch size of 5, 200 epochs and a 0.001 learning rate.

### Division into three vertebral bodies


1

**Figure 2:**
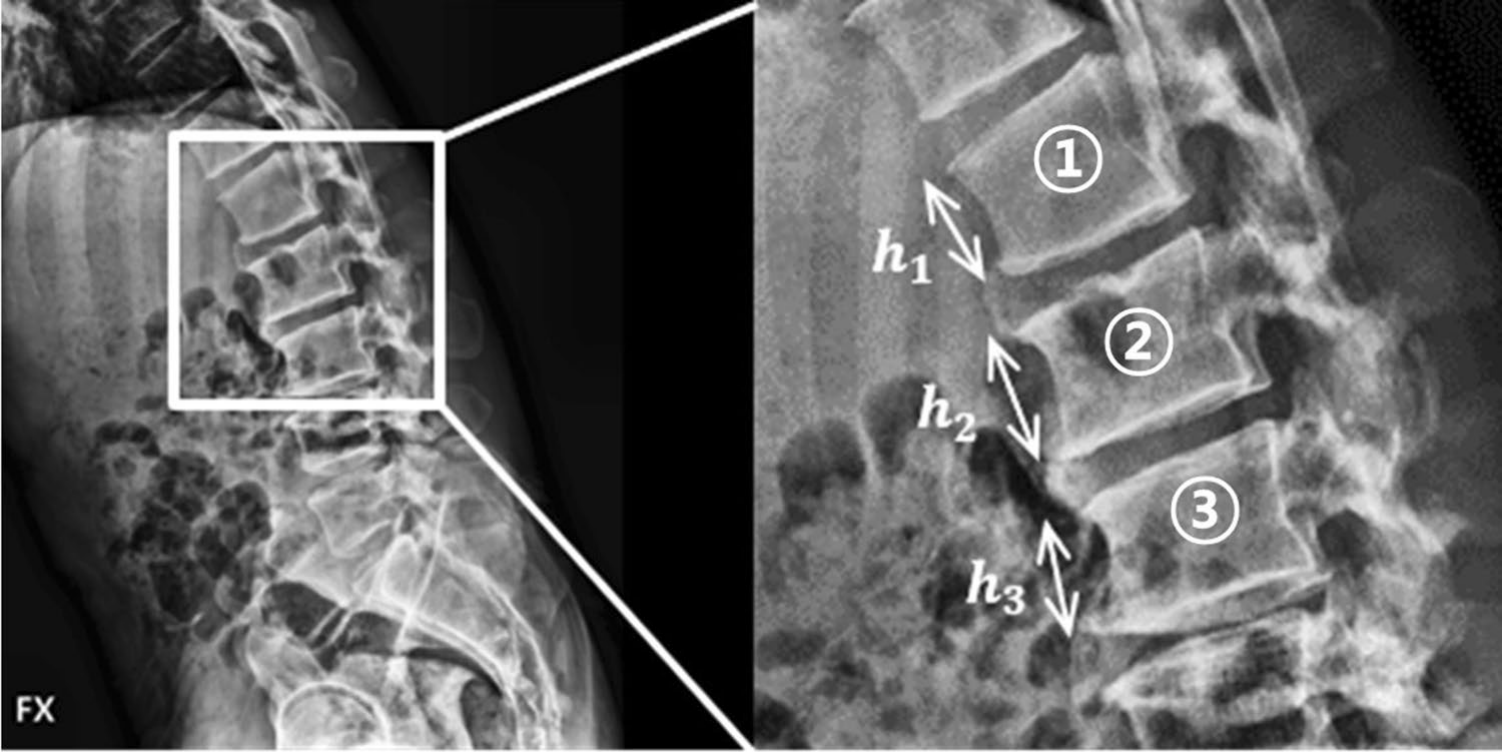
Vertebral X-ray image used for the training, and the anterior vertebral height used for calculating the vertebral compression.

**Figure 3:**
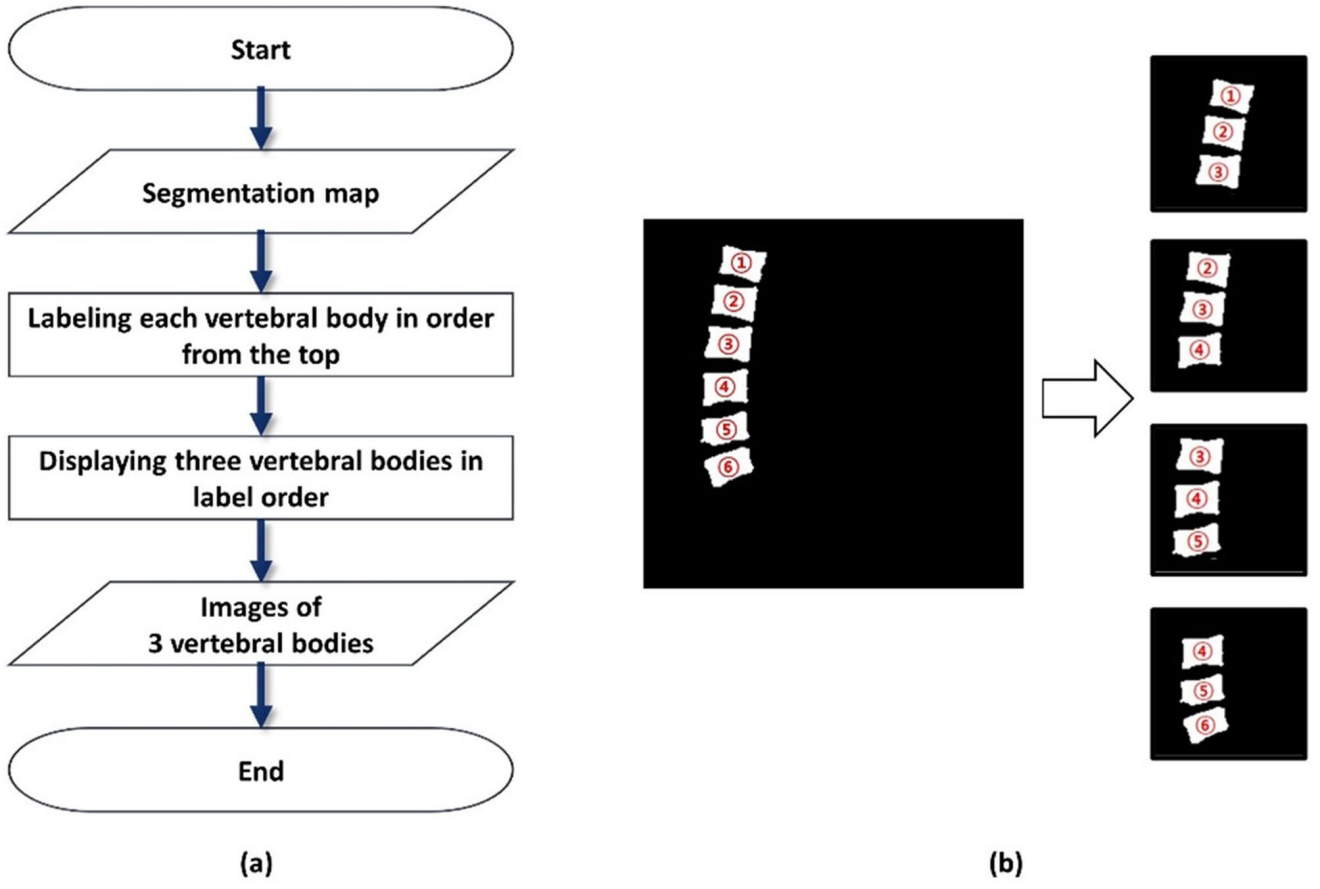
Process and images of division into three vertebral bodies from the segmentation map. (**a**) Process. (**b**) Images.

VC measurement is performed on the lateral X-ray image of the vertebrae via a process that is primarily used in clinical practice and is expressed as Eq. (1)^26,27^ (Fig. 2). A radiologist measured the VC manually, and the measurements were used as the GT for the VC measurement model. The maximum percentage value for the VCs in the data is 59.06 (%), whereas the minimum percentage value is 0.01 (%), the mean percentage value is 8.61 (%), and the standard deviation is 9.30 (%). Because the anterior heights of the upper and lower VBs adjacent to the fractured VB are required for calculating the VC for one VB, we obtained images of three VBs through the process shown in Fig. 3a. We labeled each VB in the segmented VB image from top to bottom and dividing the VB image into three units in numerical order (Fig. 3b).

### Vertebral compression measurement model based on CNN

A multi-scale residual dilated network (MRDN) was employed to measure the VC using the R2U-Net output images. The proposed MRDN model adds multi-scale residual dilated blocks (MRDBs) to the CNN-based regression model. An MRDB is composed of a bottleneck layer for computation time reduction, element-wise addition layers for residual mapping^28^, convolution filters (Conv2D), and several dilated convolution layers (Dilated Conv2D). Dilated Conv2D has multiple dilation rates (DR) for extracting features through various scales of receptive fields^29^.

**Figure 4:**
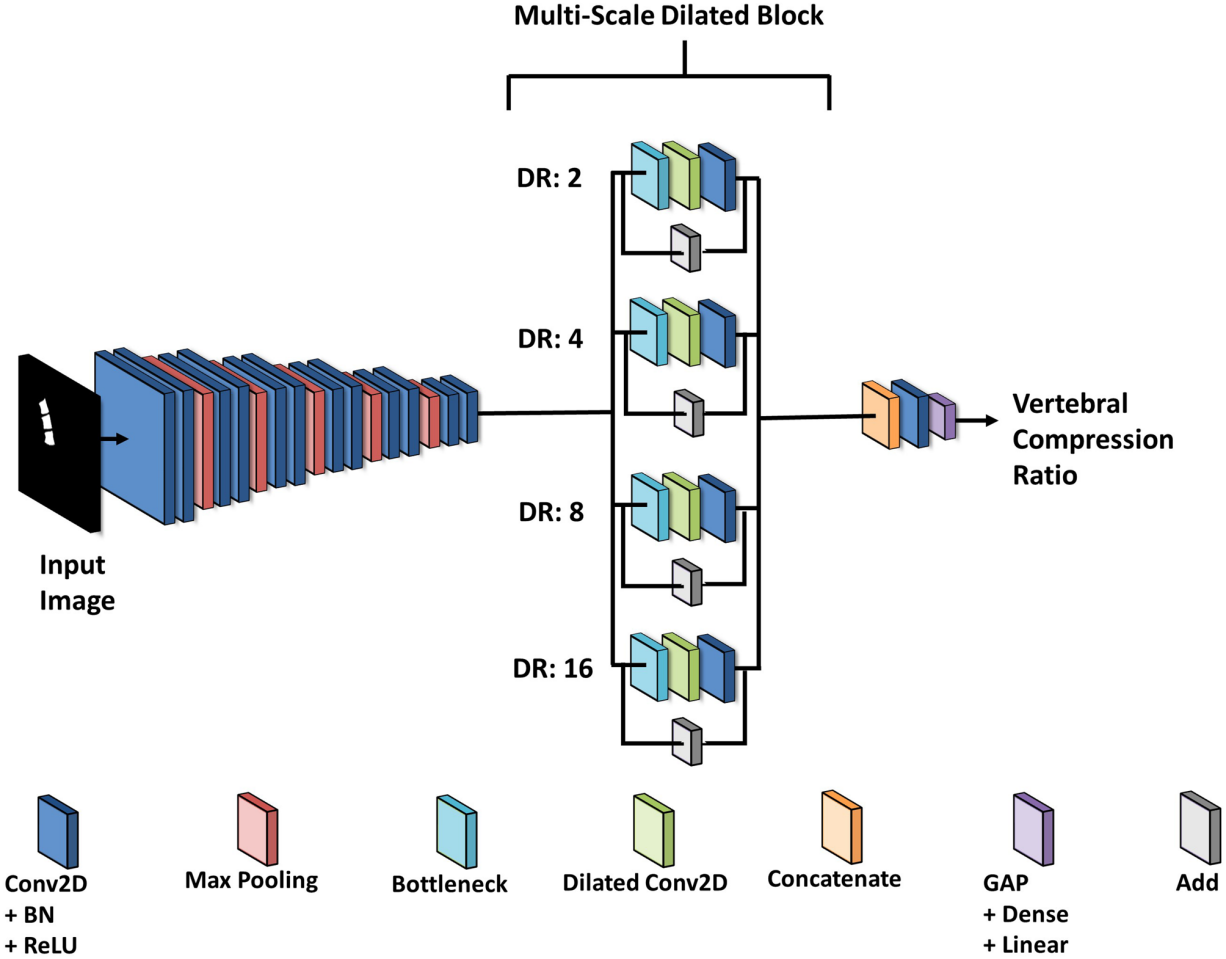
CNN-based artificial neural network model for vertebral compression measurement.

As shown in Fig. 4, six layers comprising Conv2D with a 3 × 3 kernel size and a 1 × 1 stride, BN, ReLU, and max pooling with a 2 × 2 kernel size were applied to extract a low-level feature map. Subsequently, because numerous parameters are generated, the MRDB included bottleneck layers, four Dilated Conv2D layers with 2, 4, 8, and 16 DR, Conv2D with a 3 × 3 kernel size and a 1 × 1 stride, and Add layer was inserted only in the last part of the model. Every extracted feature map from the MRDB was concatenated. Finally, the VC was measured using global average pooling (GAP) and a linear function. The model was trained for a batch size of 8, maximum 150 epochs and a 0.01 learning rate.

We have verified the performance of proposed model as comparing the performance of other CNN network models and image processing method. The selected CNN networks are ResNet50^28^, DenseNet121^30^, and CARN. ResNet consists of a skip connection and a bottle neck structure and showed excellent performance in a deep neural network through residual learning and have influenced most of the model development structures in recent years. DenseNet has a structure that reuses the features extracted from layers in the whole network. The CARN model proposed in the study most related to this paper has a structure that selectively reuses features of adjacent layers through an amplifier unit and has the advantage of alleviating overfitting through a local shape constrained manifold regularization loss function. Therefore, using our data, the models were trained, evaluated their performance and compared the results of performance.

### Vertebral compression measurement using image processing

**Figure 5:**
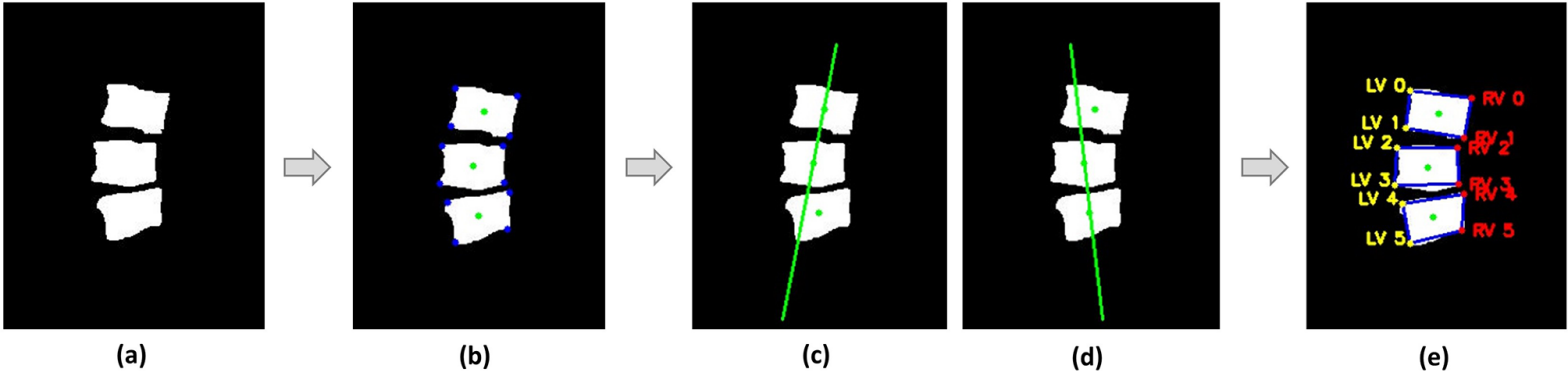
The process of obtaining the vertex location of each VB to obtain anterior VB heights. (**a**) Represents a three vertebral body image for measurement of vertebral compression. (**b**) Shows the color points. The green points indicate centers and the blue points indicate vertexes of each vertebral body. (**c**, **d**) Shows the green lines which is the criterion for separating the left and right part. (**e**) Shows the divided points to left and right. The yellow points represent left vertex and the red points represent right vertex. LV, Left vertex; RV, Right vertex.

To compare deep learning methods, we measured the VC using image processing. Figure 5 shows a process getting the vertex location of each VB to calculate anterior VB heights for deriving the VC. In the three VB from VB segmentation map (Fig. 5a), the vertexes and centers of each VB could be found (Fig. 5b). Using two points from the centers, the lines divide each of vertex to left and right part (Fig. 5c,d). Based on the lines, each vertex is defined the two parts (Fig. 5e), and the anterior VB heights could be gained using left points. Then VC was measured by applying the obtained three anterior VB heights to Eq. (1).

## Results

### Vertebral body segmentation network model

**Figure 6:**
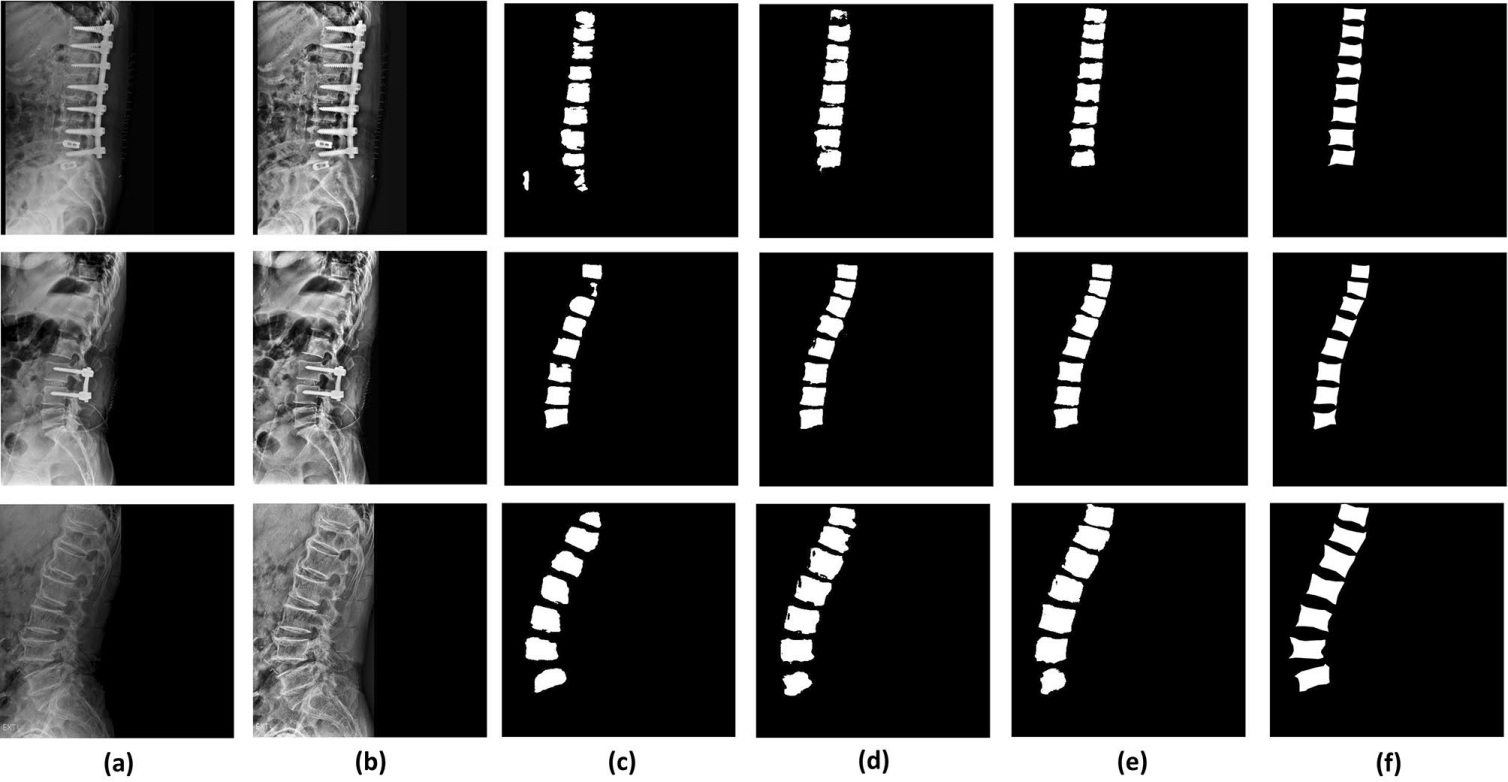
Comparison of vertebral body segmentation results based on U-Net, ResU-Net and R2U-Net model and ground truth. (**a**) Original X-ray images, (**b**) preprocessed X-ray images (**c**) segmentation results from U-Net model, (**d**) segmentation results from ResU-Net (**e**) segmentation results from R2U-Net (**f**) manually segmented vertebral bodies.

Figure 6 shows the comparison of ground truth with examples of the results of three CNN-based deep learning models (that is, U-Net, ResU-Net and R2U-Net) applied for vertebral body segmentation. Figure 6a is an example of original lateral X-ray images and Fig. 6b is a preprocessed image that not used for training Fig. 6e is a ground truth image for the X-ray image of the same row (Fig. 6a). Figure 6c–e are the segmentation result images of Fig. 6a obtained from U-Net model, ResU-Net, and R2U-net in order.

Using the models, we compared the GT region and the predicted vertebral body region (in pixel units) to calculate: true positive (TP), false positive (FP), false negative (FN), and true negative (TN). Using each value, we verified the performance on sensitivity, specificity, accuracy, and the Dice similarity coefficient (DSC) using Eqs. (2)–(5). The sensitivity refers to the probability that the model correctly predicts to vertebral body region. The specificity is the probability that the model correctly predicts to background region. The accuracy represents the probability of the model to classify each pixel correctly for all areas. The DSC is an index that measures of similarity between the predicted result from the model and ground truth and is typically used to evaluate the performance of image segmentation. The average values of the four conditional probabilities for R2U-net were: sensitivity, 0.934 (± 0.086); specificity, 0.998 (± 0.002); accuracy, 0.987 (± 0.005); and DSC, 0.923 (± 0.073) (Table 1).

2 $$Sensitivity=\frac{TP}{TP+FN}$$

3 $$Specificity=\frac{TN}{TN+FP}$$

4 $$Accuracy=\frac{TP+TN}{TP+TN+FP+FN}$$

5 $$Dice\;Similarity\;Coefficient\left( {DSC} \right) = \frac{{2TP}}{{2TP + FP + FN}}$$

**Table 1:** Comparison of performance between three deep learning model for vertebral body segmentation. R2U-net showed the highest performance in the four conditional probability values, and the values are shown in bold.

	U-net (± SD)	ResU-net (± SD)	R2U-net (± SD)
Sensitivity	0.930 (± 0.116)	0.925 (± 0.065)	**0.934 (± 0.086)**
Specificity	0.997 (± 0.001)	0.997 (± 0.003)	**0.998 (± 0.002)**
Accuracy	0.987 (± 0.006)	0.987 (± 0.005)	**0.987 (± 0.005)**
DSC	0.903 (± 0.090)	0.920 (± 0.068)	**0.923 (± 0.073)**

*SD* standard deviation, *DSC* dice similarity coefficient.

### Vertebral compression measurement model

Because the vertebral compression measurement is only performed on the fractured VB, we have evaluated a performance of the model on 83 test data with fractures that were not used for training. To evaluate the performance of the proposed model, we compared the manually measured VC and the measured results from the model. The mean absolute error (MAE), mean square error (MSE), and root mean square error (RMSE) were used to verify the model's performance (Eqs. 6–8).

6 $$MAE=\frac{1}{n}\sum _{i=1}^{n}|{x}_{i}-x|$$

7 $$MSE=\frac{1}{n}\sum _{i=1}^{n}{\left|{x}_{i}-x\right|}^{2}$$

8 $$RMSE=\sqrt{\frac{1}{n}\sum _{i=1}^{n}{|{x}_{i}-x|}^{2}}$$

where ${x}_{i}$ is the compression ratio measured manually, $x$ is the VC measured via the MRDN, and $n$ is the number of test data. Table 2 shows the MAE, MSE, and RMSE according to the difference between the manual measurement of vertebral body compression and the automatic measurement of vertebral body compression using two methods: image processing method and deep learning models based on CNN. The performance of proposed MRDN analysis yielded an average MAE of 2.637 (± 1.872), an average MSE of 13.985 (± 24.107), and an average RMSE of 3.739 (± 2.187). To evaluate the performance of the proposed model, we compared DenseNet121, ResNet50, CARN against the MRDN and image processing.

**Table 2:** Comparison of performance between methods using deep learning networks and image processing. The performance values of the proposed MRDN are shown in bold.

	Image processing	ResNet50 (± SD)	DenseNet121 (± SD)	CARN (± SD)	Proposed MRDN (± SD)
MAE (%)	5.255 (± 0.929)	4.825 (± 1.611)	4.474 (± 0.812)	3.496 (± 2.365)	**2.637 (± 1.872)**
MSE (%)	48.467 (± 31.091)	45.657 (± 19.887)	40.244 (± 10.020)	22.359 (± 29.021)	**13.985 (± 24.107)**
RMSE (%)	6.962 (± 1.515)	6.757 (± 1.541)	6.344 (± 0.751)	4.729 (± 2.934)	**3.739 (± 2.187)**

*SD* standard deviation, *MAE* mean absolute error, *MSE* mean square error, *RMSE* root mean square error.

**Figure 7:**
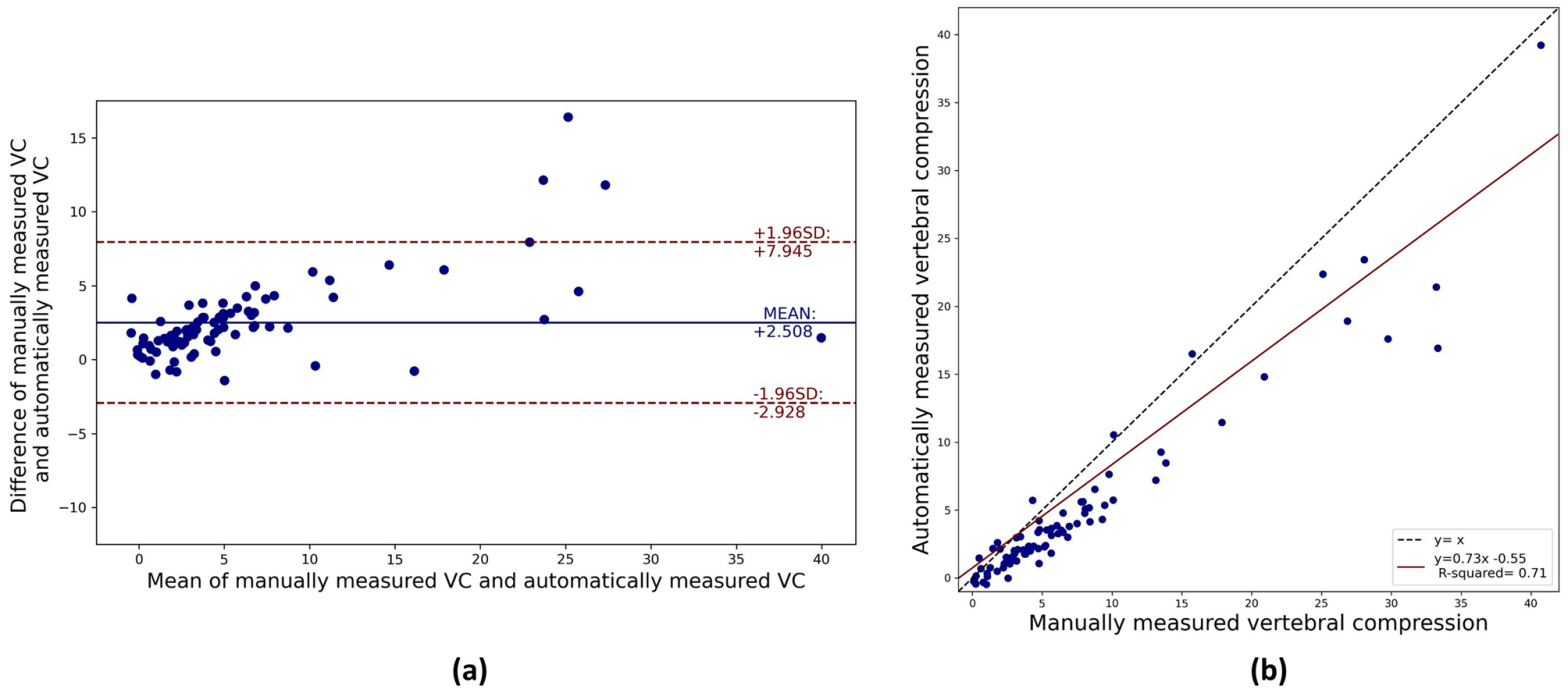
Comparing and analyzing the automatically measured VCs from the proposed model and manually measured VCs used ground truth. (**a**) Bland–Altman plot analysis automatically measured VCs and manually measured VCs, (**b**) Scatter plot of correlation between the automatically measured VCs and the manually measured VCs.

A Pearson correlation analysis of the results indicates a strong positive correlation of 0.946 (p < 0.05). Bland–Altman plot analysis was performed to compare the measured results of the proposed model against the GT. As observed in Fig. 7a, 95% of the results the results from proposed model fall within the 95% confidence interval. Figure 7b presents the scatter plot of the automatically measured VCs from the proposed model and manually measured VCs used ground truth, and the regression equation.

## Discussion

We propose an automated algorithm for directly measuring the VC in a lateral X-ray image of the spine. The algorithm consists of a R2U-Net for vertebral body segmentation and a MRDN model for VC measurement. The performances of vertebral compression measurement models are dependent on the results of segmentation. Therefore, we trained segmentation models (that is, U-Net, ResU-Net, and R2U-Net) and compared their performance. According to the comparing results, we selected R2U-Net which exhibited the best performance in DSC among other compared U-Net families. The model’s performances were evaluated by comparing the VBs obtained via the CNN model with VBs manually segmented by a radiologist. R2U-Net showed an average sensitivity of 0.934 (± 0.086), an average specificity of 0.998 (± 0.002), an average accuracy of 0.987 (± 0.005), and an average DSC of 0.923 (± 0.073), indicating accurate segmentation. The vertebral compression measurement model for deep learning-based regression analysis was trained, except for the segmented vertebral body images with inaccurate segmentation results.

We compared the results of the vertebral compression measurement method using image processing and the deep learning-based artificial intelligence model. In all methods, the error of the vertebral body with severe compression was larger than that of the vertebral body with low compression. It could be observed that the higher the severity of vertebral compression, the lower the performance of vertebral segmentation, and the measurement of vertebral compression using incorrect segmentation results increases the error rate. This is because most of our data consisted of treated by orthopedic and neurosurgery patients, so the data on severe vertebral compression were relatively insufficient for training that of data. Hence, we estimated that the imbalance of data leaded a lower performance in a range of data with severe vertebral compression. We could be found that this more effected on the measurement through the image processing method. Compared to image processing and deep learning methods, the performance of the deep learning model was significantly better. Anterior VB heights, a variable necessary to calculate VC, are measured according to the number of pixels in the segmentation results. Even though it is a result of good performance (DSC 90% or more), there is a difference from the GT segmentation map, and the difference from the manually measured VC increases when the Eq. (1) is applied to derive the VC. Especially, when the vertexes of the VB were not clearly visible, or in the case of a VB with severe osteoporotic VC, the wrong points were selected during the vertex selection process (Fig. 5). In this case, the error was very large. On the other hand, because the deep learning model extracts the features of the entire image and considers the relationship between the segmentation result and the manual measurement value, we predicted that the measurement methods using deep learning showed relatively high performance. Therefore, it is assumed that higher accuracy performance can be expected by performing learning by adding enough data of patients with severe vertebral compression in the future.

The performance of the proposed MRDN was evaluated by comparing the VC obtained via the trained model with the VC measured manually by a radiologist, and the evaluation results were: MAE, 2.637 (± 1.872); MSE, 13.985 (± 24.107); and RMSE, 3.739 (± 2.187). From the Pearson correlation analysis, we found a positive correlation of 0.95 at p < 0.05. We trained DenseNet121, ResNet50, and CARN models for performance comparison with the proposed model. From the performance comparison, the average MAE for the ResNet50 model was 4.825 (± 1.611), 4.474 (± 0.812) for DenseNet121, and 3.496 (± 2.365) for CARN. The data used in the VC measurement process is a segmented map image. Therefore, we estimated that the excessively many parameters did not have a significant effect on measurement of VC, and that CARN and MRDN with relatively shallow depth structure than ResNet50 and DenseNet121 showed higher performance comparing them. Moreover, as comparing to MRDN and CARN, MRDN exhibited higher performance than CARN. This indicates that the receptive fields of various scales through the MRDBs were advantageous for extracting the features of the correlation between the three VBs and the VC. We calculated the compression of all normal and fractured VB. When this calculation is applied to a normal VB adjacent to a vertebral body in which the height of the anterior vertebral body is lost, it can be calculated as a negative value. Therefore, only clinically significant fractured VB were newly evaluated, and the results are shown in Fig. 7 and Table 2. However, the negative data were used for training process, it seems to make throughout the results to be measured lower than the manual measurement.

In the future, the performance is expected to improve if a superior preprocessing scheme is added and more data, especially fractured severe compression vertebral data, are collected for the training. In addition, because vertebral compression fracture diagnostic indicators include Cobb angle, intervertebral disc height loss, and VC, further studies on measuring these indicators are expected to improve the accuracy of a diagnosis of vertebral compression fractures by assisting in the interpretation of the images. Furthermore, applying this algorithm to the medical picture archiving and communication systems is more practical than directly measuring VC manually as is currently done in the medical field.

## Supplementary Information

Supplementary Figure 1.

## Data Availability

The X-ray image data used to support the findings of this study are available upon request from the corresponding author.
