# Author Correction: A deep learning algorithm for automated measurement of vertebral body compression from X-ray images

**DOI:** 10.1038/s41598-022-10034-0

**Published:** 2022-04-04

**Authors:** Jae Won Seo, Sang Heon Lim, Jin Gyo Jeong, Young Jae Kim, Kwang Gi Kim, Ji Young Jeon

**Affiliations:** 1 Department of Health Sciences and Technology, GAIHST, Gachon University, Incheon, 21999 Korea; 2 Department of Biomedical Engineering, Gachon University College of Medicine, 38-13 Docjeom-ro 3-bungil, Namdong-gu, Incheon, 21565 South Korea; 3 Department of Radiology, Gachon University College of Medicine, Gil Medical Center, 38-13 Docjeom-ro 3-bungil, Namdong-gu, Incheon, 21565 South Korea

Correction to: *Scientific Reports* 10.1038/s41598-021-93017-x, published on 2 July 2021

The original version of this Article contained an error in Affiliation 1, which was incorrectly given as ‘Department of Health Sciences and Technology, Gachon Advanced Institute for Health Sciences and Technology (GAIHST), Gachon University, Seongnam-si 13120, South Korea’. The correct affiliation is listed below.

Department of Health Sciences and Technology, GAIHST, Gachon University, Incheon 21999, Korea.

Additionally, this Article contained an error in the Acknowledgments section.

"This work was supported by Institute of Information & Communications Technology Planning & Evaluation (IITP) Grant funded by the Korea Government (MSIT) (No. 2020-0-00161-001, Active Machine Learning based on Open-set training for Surgical Video), and the GRRC program of Gyeonggi province. [GRRC-Gachon2020(B01), AI-based Medical Image Analysis], and the Gachon Program (GCU-202008440010)."

now reads:

"This work was supported by Institute of Information & Communications Technology Planning & Evaluation (IITP) Grant funded by the Korea Government (MSIT) (No. 2020-0-00161-001, Active Machine Learning based on Open-set training for Surgical Video), and the GRRC program of Gyeonggi province. [GRRC-Gachon2020(B01)], AI-based Medical Image Analysis], and the Gachon Program (GCU-202106290001)."

The original Article has been corrected.

