# Supplementary material for: A deep learning algorithm for automated measurement of vertebral body compression from X-ray images
Source: Sci Rep. 2021 Jul 2;11:13732. doi: 10.1038/s41598-021-93017-x (PMC8253748; doi:10.1038/s41598-021-93017-x)
Supplement: Supplementary file 1 — Supplementary Figure 1. [file 41598_2021_93017_MOESM1_ESM.pdf]

A deep learning algorithm for automated measurement of vertebral body compression from X-ray images

Jae Won Seo[1], Sang Heon Lim[1], Jin Gyo Jeong[1], Young Jae Kim[2], Kwang Gi Kim[1,2,*], and Ji Young Jeon[3*]

[1] Department of Health Sciences and Technology, Gachon Advanced Institute for Health Sciences and Technology (GAIHST), Gachon University, Seongnam-si 13120, Korea.

[2] Department of Biomedical Engineering, Gachon University College of Medicine, 38-13 Docjeom-ro 3-bungil, Namdong-gu, Incheon, 21565, Korea.

[3] Department of Radiology, Gachon University College of Medicine, Gil Medical Center, 38-13 Docjeom-ro 3-bungil, Namdong-gu, Incheon, 21565, Korea.

Kwang Gi Kim & Ji Young Jeon equally contributed to this work as corresponding author.

* Co-correspondence to Kwang Gi Kim; E-mail: kimkg@gachon.ac.kr & Ji Young Jeon; E-mail: mdjeonjy@gilhospital.com

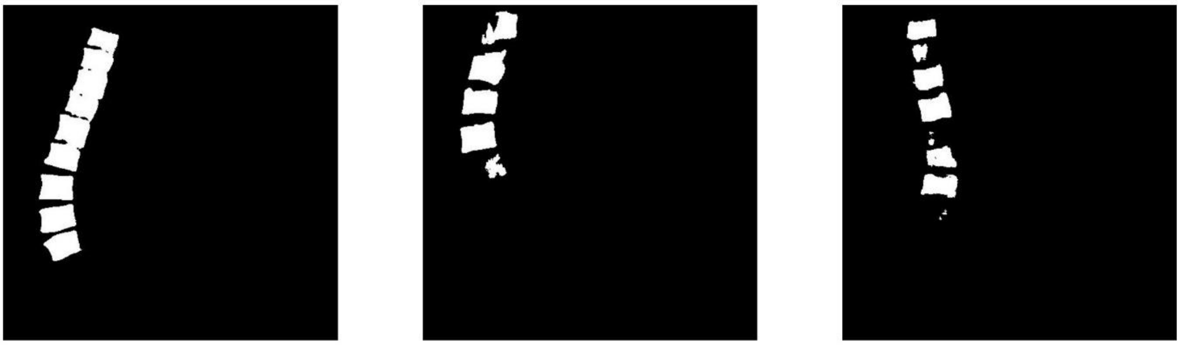

(a) (b) (c)

Supplementary Figure 1. examples of results of inaccurate segmentation results. (a) each vertebral body couldn't be subdivided into one (b) the case that the corners of one vertebral body couldn't be able to be found (c) The case that the vertebral bodies are not continuous.
